# Cost-effectiveness analysis of whole-genome sequencing during an outbreak of carbapenem-resistant *Acinetobacter baumannii*

**DOI:** 10.1017/ash.2021.233

**Published:** 2021-12-13

**Authors:** Thomas M. Elliott, Patrick N. Harris, Leah W. Roberts, Michelle Doidge, Trish Hurst, Krispin Hajkowicz, Brian Forde, David L. Paterson, Louisa G. Gordon

**Affiliations:** 1 QIMR Berghofer Medical Research Institute, Herston, Brisbane, Queensland, Australia; 2 The University of Queensland, Centre for Clinical Research, Herston, Brisbane, Queensland, Australia; 3 Central Microbiology, Pathology Queensland, Royal Brisbane and Women’s Hospital, Herston, Brisbane, Queensland, Australia; 4 School of Chemistry and Molecular Biosciences, University of Queensland, Brisbane, Queensland, Australia; 5 European Bioinformatics Institute (EMBL-EBI), Wellcome Genome Campus, Hinxton, United Kingdom; 6 Infectious Diseases Unit, Royal Brisbane and Womens’ Hospital, Herston, Brisbane, Queensland, Australia; 7 The University of Queensland, School of Public Health, Brisbane, Queensland, Australia; 8 Queensland University of Technology, School of Nursing, Kelvin Grove, Brisbane, Queensland, Australia

## Abstract

**Background:**

Whole-genome sequencing (WGS) shotgun metagenomics (metagenomics) attempts to sequence the entire genetic content straight from the sample. Diagnostic advantages lie in the ability to detect unsuspected, uncultivatable, or very slow-growing organisms.

**Objective:**

To evaluate the clinical and economic effects of using WGS and metagenomics for outbreak management in a large metropolitan hospital.

**Design:**

Cost-effectiveness study.

**Setting:**

Intensive care unit and burn unit of large metropolitan hospital.

**Patients:**

Simulated intensive care unit and burn unit patients.

**Methods:**

We built a complex simulation model to estimate pathogen transmission, associated hospital costs, and quality-adjusted life years (QALYs) during a 32-month outbreak of carbapenem-resistant *Acinetobacter baumannii* (CRAB). Model parameters were determined using microbiology surveillance data, genome sequencing results, hospital admission databases, and local clinical knowledge. The model was calibrated to the actual pathogen spread within the intensive care unit and burn unit (scenario 1) and compared with early use of WGS (scenario 2) and early use of WGS and metagenomics (scenario 3) to determine their respective cost-effectiveness. Sensitivity analyses were performed to address model uncertainty.

**Results:**

On average compared with scenario 1, scenario 2 resulted in 14 fewer patients with CRAB, 59 additional QALYs, and $75,099 cost savings. Scenario 3, compared with scenario 1, resulted in 18 fewer patients with CRAB, 74 additional QALYs, and $93,822 in hospital cost savings. The likelihoods that scenario 2 and scenario 3 were cost-effective were 57% and 60%, respectively.

**Conclusions:**

The use of WGS and metagenomics in infection control processes were predicted to produce favorable economic and clinical outcomes.

*Acinetobacter baumannii* is an opportunistic pathogen commonly associated with bacteremia, pneumonia, and urinary tract infections in patients admitted to intensive care units (ICUs) and burn units.^1^ The persistence of *A. baumannii* in hospital environments, and the ability of the organism to readily acquire or develop resistance to a wide range of antibiotics, has led to high rates of carbapenem-resistant *A. baumannii* (CRAB), a frequent cause of hospital outbreaks,^2^ with rates reportedly 2- to 5-fold higher in ICUs.^3^ Aqueous reservoirs, such as drains, sinks and toilets, have been identified as a pathway for spreading *A*. *baumannii* to patients.^4^ Approximately 5%–30% of surfaces can remain contaminated because existing detergent and disinfectant formulations cannot disrupt biofilms.^5^ An enhanced focus on cleaning practices have been shown to be effective at reducing contamination and are cost-effective.^6^ Identifying environmental contamination early in outbreaks is critical to limiting their spread.

Hospital infection control teams use microbiological screening to identify pathogens, their susceptibility in patients and for environmental screening. This process takes 1–3 days for bacteria that are cultured on routine media, with antimicrobial susceptibility testing adding 1–2 days.^7^ Whole-genome sequencing (WGS) has higher precision compared with conventional typing methods and is used to detect outbreaks,^8^ monitor the evolution of drug resistance,^9^ and reconstruct transmission routes.^10^ WGS requires the sample to be cultured beforehand, whereas WGS shotgun metagenomics (metagenomics) attempts to sequence the entire genetic content straight from the sample. Metagenomics can characterize the subtypes, antimicrobial resistance, and pathogenic gene carriage of the microbial population.^11^ Turnaround times for metagenomics range from 2 to 7 days from sample collection to results.^12^ Because time to diagnosis is not always shortened, the diagnostic advantages lie in the ability to detect unsuspected, uncultivatable, or very slow-growing organisms, which produce negative results with standard assays.^7^ The overwhelming amount of host DNA present in primary clinical specimens collected from patients poses a major challenge in metagenomics, but this is not an issue when it is used on environmental samples.^7^

The importance of WGS in limiting multidrug-resistant organism (MDRO) nosocomial infections has been identified in outbreak^13^ and nonoutbreak settings.^14^ Significant cost savings could be realized as the reduction in cases, and subsequent resources used for contact precautions, outweigh the additional cost of sequencing,^13–15^ but more evidence on the economic impact of WGS within infection control is needed. To our knowledge, the economic impact of metagenomics has not been studied, specifically in environmental contamination screening.

To address this gap, we performed a cost-effectiveness analysis of WGS and metagenomics of environmental samples to inform future resource use of WGS based on a hospital CRAB outbreak.

## Methods

In the intensive care and burn units of a large metropolitan hospital in Brisbane, Australia, we used a hybrid agent-based and discrete-event simulation model to assess the management of a CRAB outbreak, and we incorporated background MDRO nosocomial infections and environmental screening. This study was approved by the QIMR Berghofer Medical Research Institute Human Research Ethics Committee (P2353) and the Queensland Government Public Health Act Human Research Ethics Committee (RD007427).

### The outbreak

**Fig. 1.**
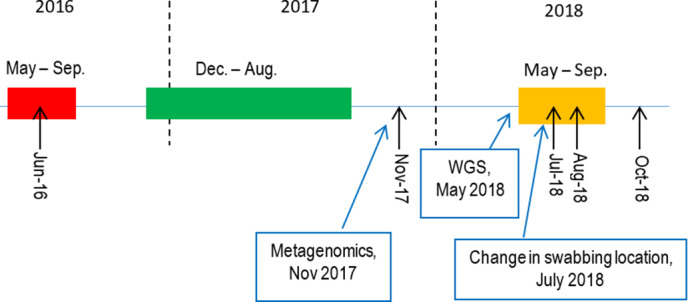
Outbreak timeline with investigation strategy changes. Each colored box represents a distinct outbreak of ST1050 CRAB. The red box consisted of 17 cases, the green box represents 8 cases and the orange box represents 11 cases. Each black arrow indicated initiation of environmental screening. To improve specimen collection the focus of environmental swabbing changed from high-touch areas to high bacterial load areas in July 2018. High-touch areas were defined as places commonly touched such as nurse keyboards, trolleys and door handles. High bacterial-load areas were defined as areas of high biomass such as floor drains, plumbing and inside burns bath drains. WGS was implemented as part of outbreak control in May 2018 and metagenomics in November of 2017. Note. WGS, whole-genome sequencing.

The hospital is a 978-bed, tertiary-care facility in Queensland, Australia, comprised of open-ward, 4-bed, 2-bed, and single-bed accommodation combinations. The outbreak predominantly affected a 34-bed ICU and an 18-bed burn unit over a 32-month period. Active transmission of 17 cases of sequence type (ST) 1050 (ST1050) CRAB were identified between May and August 2016; 6 cases were identified between December 2016 and August 2017; and 8 cases were identified between May and August 2018 (Fig. 1).^16^ Monthly WGS reporting starting in June 2016 identified environmental contamination as the likely source of the ongoing outbreak. Environmental metagenomics was introduced in November 2017 but had reduced sensitivity due to low DNA yields from sampling. Instead, areas of high bacterial load, such as drains and burn baths, were targeted in 2018, which revealed 4 areas positive for CRAB. Immediate reporting of WGS results were available when the CRAB outbreak resurfaced in May 2018. Additional details about the outbreak, implementation of metagenomics, environmental swabbing, and sequencing outcomes are discussed in the Supplementary Materials and by Roberts et al.^16^

### Comparison groups

We evaluated the cost-effectiveness of 3 screening scenarios as follows:

- Scenario 1: The observed outbreak described above with initially no WGS, no metagenomics and environmental swabbing concentrating on high touch areas. WGS, metagenomics and environmental swabbing of high load areas were introduced later as described above (Fig. 1).
- Scenario 2: Hypothetically testing immediate WGS use prior to the start of the outbreak. This would lead to identifying the need for environmental swabbing to focus on high bacterial load areas. As in Scenario 1, metagenomics was introduced in November 2017.
- Scenario 3: Hypothetically testing WGS and metagenomics use prior to the start of the outbreak.

### Model structure

Using AnyLogic dynamic simulation modeling software (AnyLogic, Chicago, IL), the evaluation combined methods of cost-effectiveness analysis,^17^ infectious disease modeling and system dynamics.^18^ A network approach was taken to connect the hospital environment, the dynamics of pathogen transmission, environmental pathogen contamination, patient movements, and decisions by the hospital infection control team. The intensive care and burns units were modeled; 29 of the 31 ST1050 CRAB cases were detected in these units. The model ran for 32 months (January 4, 2016, through January 1, 2019) in hourly units. The spread of CRAB was calibrated to outbreak data to obtain both patient and environmental transmission rates (see Supplementary Material, section ST1050 CRAB spread’). The spread of other MDROs through the intensive care and burns units were based on surveillance data between April 2016 and January 2019. The 3 main interacting components in the model were patient flow dynamics, pathogen transmission dynamics, and outbreak control team decisions. Full model details are provided in the Supplementary Materials (detailed description of model structure).

**Fig. 2.**
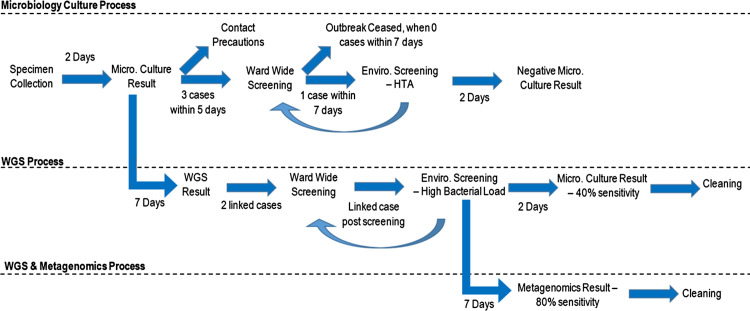
Introduction of WGS and metagenomics into microbiology culture infection control process. Note. micro, microbiology; enviro, environmental; WGS, whole-genome sequencing; HTA, high-touch area.

Pathogen transmission dynamics were modeled daily at the ward level through patient-to-patient transmission and contaminated room-to-patient transmission using a discrete-time event model. The daily probability a susceptible patient was colonised/infected was calculated using the frequency-dependent transmission formula ( 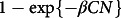 ).^19^ We modeled new daily colonization and/or infections with the binomial distribution formula ( 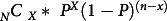 ), where *x* is the number of transmissions (limited to 3), *n* is the number of susceptible patients, and *P* is the probability. Daily incident probabilities of other MDROs detected were included, and information on cluster spread was derived from the WGS results (Supplementary Table S2). Decisions by the infection control team, like outbreak designation and initiating environmental screening, were initially based on microbiological cultures before transitioning to WGS and metagenomics information (Fig. 2).

**Fig. 3.**
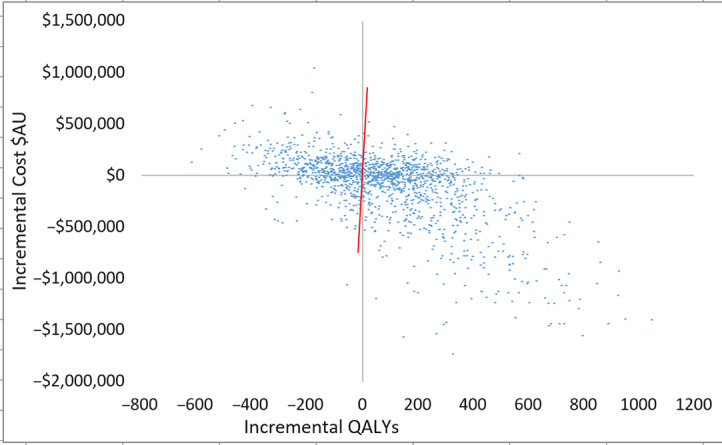
Scatterplot of incremental costs and QALYs (all patients) for scenario 3 versus scenario 1. Each dot represents an incremental cost and incremental QALY pairing, using the assigned distributions around each model parameter, selected randomly during 5,000 iterations. Dots falling below the diagonal line (the willingness-to-pay threshold of AU$50,000 per QALY) are considered cost-effective. The proportion of simulations considered cost-effective was 60.1%. Note: QALYs, quality-adjusted life years.

### Model parameters

Patient hospital episode information was obtained from the hospital-based corporate information system (HBCIS) for all patients who spent time in either the intensive care or burns units between April 1, 2016, and January 1, 2019. HBCIS routinely collects all patient separations and patient days (or occupied bed days) that occur in public hospitals. Hospital daily admission rate, ward admission probability, ward transfer proportions, and ward length of stay were estimated empirically from the HBICS data set (Supplementary Tables S4–S8). Ward stays were estimated as independent γ distributions for all observed ward pair combinations using the methods of moments approach.^20^

The sensitivity of detecting environmental CRAB contamination with microbiology cultures was estimated at 40% and metagenomics at 80% (Table 1). These estimates were calculated from 5 positive environmental samples from 50 sequenced samples. Due to the uncertainty of samples not being detected, these values were varied in sensitivity analysis (Table 1).

**Table 1.** Parameter Description, Values, and Sources Used in the Hybrid Simulation Model

Parameter	Value (Sensitivity Analysis^a^)	Source
**Initialization**		
Initial starting population, no.	106	HBCIS data
Ward	Burns, ICU, ID, other, readmission	Building floor plans
Population entry rate, average patients per day	7.40	HBCIS data
Ward admission, transfers and LOS	see Supplementary Tables S4–S8	HBCIS data
**Outbreak spread**		
Transmission patient parameter 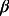 _1_	0.65	Calibration
Transmission patient parameter 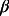 _2_	0.014	Calibration
Transmission env. parameter 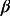 _1_	0.045	Calibration
Transmission env. parameter 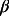 _2_	0.019	Calibration
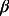 _1_ to 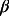 _2_ change date	1/10/2016	Assumption based on outbreak data
**Infection control**		
Microbiology test processing time, d	2	Clinical data
Genome sequencing processing time, d (range)	7 (3–10)	Clinical data
Outbreak trigger test	3 cases in 5 days	Clinical data
Outbreak ceases	0 cases within 7 days	Clinical data
Environmental swab test	1+ cases in week after screening	
Prob. effective swab returns positive result when contaminated^d^	0.40 (0.34, 0.46)^a^ (α = 189.23, β = 283.84)^2^	Clinical data
Prob. metagenomics returns positive result when contaminated^d^	0.80 (0.68, 0.92)^a^ (α = 252.04, β = 63.01)^b^	Clinical data
**Environmental contamination**		
Prob. of contaminating HTA of single bed	0.14	Ng 2018,^37^ Lerner 2020^38^
Prob. of contaminating HTA of multi bed	0.09	Ng 2018,^37^ Lerner 2020^38^
Prob. of contaminating drainage of en suite bathroom	0.17	Calibration
**Costs**		
Microbiology test cost, AU$	$82	Elliott (2020)^13^
WGS cost, AU$	$150 (120,180)^a^ (α = 44.44, β = 3.38)^c^	Study cost
Basic bedroom cleaning cost, AU$	$70	Elliott (2020)^13^
Advanced bedroom cleaning cost, AU$	$140	Assumption of double basic cost
Bed closure, AU$	$231	Page (2017)^26^
Bed closure in ICU, AU$	$466	Page (2017)^26^
Environmental screening swab, AU$	$3	Fishersci (2020)^29^
Environmental screening culture, AU$	$34	Elliott (2020)^13^
Metagenomics, AU$^e^	$355 (251,459)^a^ (α = 44.44, β = 7.99)^c^	Ace Sequencing quote
PPE, AU$	$52	Otter (2016)^28^
**CRAB infection parameters**		
Sepsis rate	0.77	AGAR report
RTI rate	0.50	ICD-10 (OGN)(S) (Table S3)
UTI rate	0.27	ICD-10 (OGN)(R) (Table S3)
Mortality rate	0.08 (0.06, 0.15)^a^ (α = 10.73, β = 128.65)^b^	ICD-10 (OGN)(U) (Table S3)
**Cost of antibiotic treatment per patient, AU$**		
CRAB (colistin + tigecycline^f^ or colistin + meropenem^g^)	$3,199	Viehman (2014)^27^ and hospital pharmacy pricing
**Health utility values** ^h^		
Sepsis infection	0.530	Lee (2010)^23^
Respiratory infection	0.580	Lee (2010)^23^
Urinary tract infection	0.730	Lee (2010)^23^
Non infected patient	0.820	White (2020)^6^
Isolated patient multiplicative reduction factor	0.895	Mac (2019)^24^

Note. ICU, intensive care unit; SD, standard deviation; LOS, length of stay; Prob, probability; HTA, high-touch area; PPE, personal protective equipment; HBCIS, hospital-based corporate information system; ID, infectious disease; WGS, whole-genome sequencing; CRAB, carbapenem-resistant *Acinetobacter baumannii*.

a These values were used in one-way sensitivity analysis.

b These values were used for a β distribution in the probabilistic sensitivity analysis.

c These values were used for a γ distribution in the probabilistic sensitivity analysis.

d These values were calculated from 5 positive environmental samples (4 of 5 by metagenomics and 2 of 5 by culture) out of 50 total sequenced samples.

e Metagenomics cost entails $25 for a DNA extraction, $40 for the library preparation, $190 for sequencing and $100 for bioinformatics.

f Colistin administered at 275 mg for 14 d and tigecycline administered at 100 mg followed by 50 mg every 12 h for 14 d.

g Colistin administered at 275 mg for 14 d and meropenem administered at 1.0–2 g 3 times daily for 14 d.

h Patient health utility was assumed to change for 14 d after diagnosis, unless leaving hospital first.

The daily incidences of common MDROs were extracted from 3 years (April 2016 to January 2019) of MDRO surveillance data and converted to probabilities (Supplementary Table S2). ICD-10 codes from the HBICS data set identified the bloodstream, respiratory, and urinary tract infection rates for each of the MDROs (Table S3). The frequency of deaths in hospital from patients infected with any of the MDROs were obtained from published reports^21^ and ranged from 0.7% for *Clostridioides difficile* infection to 36.6% for vancomycin-resistant *Enterococcus* (Table S2).

Genetic relatedness was determined by examining the number of core-genome single-nucleotide polymorphisms (SNPs) that differ between any 2 isolates (pairwise core-genome SNP distance). Genetically related isolates were subdivided into clusters when the SNP distances between them were under a 5 SNPs per megabase threshold.^22^ Two years (December 2017 to December 2019) of processed MDRO WGS results identified clusters of extended spectrum β-lactamase (ESBL)–producing *Escherichia coli* and ESBL-producing *Klebsiella pneumoniae*.

Health utilities are cardinal values that represent the strength of an individual’s preferences for specific health-related outcomes. They are scored on a scale between 0, worst health to 1, perfect health. Health utilities are used to calculate QALYs, a measure of patient benefit, where the length of time in a health state is adjusted to reflect the quality of life (health utility score). Health utilities were used in the model to estimate the health impact of sepsis (0.53), urinary tract infection (0.73), respiratory tract infection (0.58), and an uninfected health state (0.82)^6,23^ (Table 1). We applied a negative health utility when patients were isolated.^24^

Healthcare costs, which were calculated in 2020 Australian dollars (1 AU$ = 0.68 USD^25^), were assigned to WGS (AU$150, US$102), metagenomics (AU$355, US$241), microbiology culture tests (AU$82, US$56), cleaning (AU$70/AU$140, US$48/US$95), closed bed days (AU$216/AU$466, US$147/US$317), personal protective equipment (PPE) (AU$52, US$35), environmental screening (AU$3, US$2) and antibiotic treatments (AU$176–AU$4,585, US$120-US$3,118) (Table 1 and Supplementary Table S3).^26–29^ The WGS and metagenomics costs comprised of sequencing and bioinformatics costs. Metagenomics was more expensive than WGS due to samples having ∼5 times more genetic content.

### Analysis

The main outcomes were number of MDRO cases, hospital costs, and QALYs. Model outcomes were aggregated from events that emerged from the interacting processes of ‘patient flow dynamics,’ ‘pathogen transmission dynamics,’ and ‘outbreak control team decisions.’ These outcomes were averaged >5,000 stochastic model simulations and presented as means and interquartile range (IQRs). Future costs and QALYs were discounted at 5% per year to provide present values. Incremental cost-effectiveness ratios were calculated as the difference in costs between 2 groups divided by the difference in QALYs.^17^ Probabilistic sensitivity analyses were undertaken to assess the likelihood of the scenario being cost-effective, considered at a willingness-to-pay threshold of AU$50,000 (US$34,000) per QALY gain and AU$28,033 (US$19,062) per QALY gain. One-way sensitively analyses were performed on the costs of WGS, cost of metagenomics, mortality rate of CRAB, WGS turnaround time, and the sensitivity of environmental swab culture and metagenomics. Outbreak fadeouts are a hazard of stochastic simulations; therefore, a supplementary analysis was performed which only included simulations where a CRAB outbreak occurred. Access to the full working model is available via the AnyLogic cloud at https://cloud.anylogic.com/model/72981523-81b1-4f31-b152-06bc9a906b7f?mode=SETTINGS.

## Results

In scenario 1, the infection control team detected on average 30 (IQR, 3–39) patients with CRAB, accrued total hospital costs of AU$1,608,571 (US$1,093,828) (IQR, AU$1,421,564, AU$1,677,308; US$966,664, US$1,140,569) and 6,578 QALYs (IQR, 6,476, 6,707) (Table 2). This compares with Scenario 2 outcomes of 14 fewer patients with CRAB, 59 additional QALYs and AU$75,099 (US$51,067) cost savings. When scenario 3 was compared with scenario 1, there were 18 fewer patients with CRAB, 74 additional QALYs and AU$93,822 (US$63,799) cost savings (Table 2). Both scenario 2 and scenario 3 were cost saving and improved patient QALYs compared with scenario 1. The increase in QALYs was primarily driven by increases in patient quality of life.

**Table 2.** Projected Health and Economic Outcomes Over the Outbreak by Scenario

Variable	Scenario 1 (Calibrated), Mean (IQR)	Scenario 2 (WGS), Mean (IQR)	Scenario 3 (WGS & Metagenomics), Mean (IQR)	S1 vs S2, Mean Diff (%)	S1 vs S3, Mean Diff (%)	S2 vs S3, Mean Diff (%)
**No. infections and colonizations**						
CRAB ST1050	30 (3–39)	15 (3, 18)	11 (3–13)	−14 (−49)	−18 (−62%)	−4 (−25%)
Microbiology costs, AU$^a,b^	$1,067,165 ($1,055,634–$1,080,028)	$1,104,117 ($1,082,526–$1,121,067)	$1,099,269 ($1,082,536–$1,112,690)	$36,952 (3)	$32,104 (3)	$−4,848 (0%)
Enviro. sampling costs, AU$^c^	$1,005 ($0–$147)	$1,884 ($39–$2,171)	$105 ($6–$137)	$879 (87)	$−900 (−90)	$−1,779 (−94)
Metagenomics costs, AU$	$15,905 ($0–$13,552)	$3,642 ($0–$0)	$13,343 ($605–$17,195)	$−12,262 (−77)	$−2,561 (−16)	$9,701 (266)
WGS costs, AU$^b^	$5,178 ($4,267–$5,947)	$23,146 ($19,990–$25,793)	$22,642 ($19,747–$25,184)	$17,968 (347)	$17,464 (337)	$−505 (−2)
PPE costs, AU$^b^	$287,596 ($242,741–$316,662)	$255,594 ($228,459–$275,694)	$248,919 ($226,492–$267,233)	$−32,002 (−11)	$−38,678 (−13)	$−6,675 (−3)
Other costs, AU$^b^,^d^	$49,578 ($210–$3,494)	$6,910 ($210–$2,064)	$4,848 ($210–$1,540)	$−42,669 (−86)	$−44,730 (−90)	$−2,062 (−30)
**Treatment costs, AU$**						
CRAB ST1050	$91,217 ($9,597–$139,291)	$46,977 ($9,597–$69,654)	$35,047 ($9,597–$47,985)	$−44,240 (−48)	$−56,171 (−62)	$−11,931 (−25)
Total hospital costs, AU$^b^	$1,608,571 ($1,421,564–$1,677,308)	$1,533,471 ($1,437,392–$1,586,882)	$1,514,748 ($1,439,823–$1,557,703)	$−75,099 (−5)	$−93,822 (−6)	$−18,723 (−1)
QALYs	6,578 (6,476–6,707)	6,637 (6,543–6,738)	6,652 (6,566–6,744)	59 (1)	74 (1)	15 (0%)

Note. IQR, interquartile range; WGS, whole-genome sequencing; CRAB, carbapenem-resistant *Acinetobacter baumannii*; ST1050, subtype 1050; Q, quartile; enviro, environmental; MDROs, multdrug-resistant organisms; QALYs, quality-adjusted life years; PPE, personal protective equipment; diff, difference; PCR, polymerase chain reaction assay.

a Microbiological culture and PCR.

b Costs attributed to MDROs arising in addition to the ST1050 CRAB outbreak were included; however, no differences were observed across scenarios.

c Environmental swabs and microbiological cultures.

d Cleaning costs and closed bed-day costs.

Microbiology culture tests were a major driver of total costs with 66.3%, 72.0%, and 72.6% of total costs for scenario 1, scenario 2 and scenario 3, respectively (Supplementary Fig. S2). Metagenomics and WGS are relatively small fractions of total hospital costs (∼<2%).

Plots of incremental cost-effectiveness ratios identified 58% (scenario 2 vs 1), 60% (scenario 3 vs 1) and 53% (scenario 2 vs 3) of iterations were cost-effective at AU$50,000 per QALY (Fig. 2). This changed to 57% (scenario 2 vs 1), 60% (scenario 3 vs 1), and 52% (scenario 2 vs 3) of iterations at willingness-to-pay threshold of AU$28,033 per QALY. QALYs and total costs both decreased in ∼13% of iterations. Cost savings were identified in 50% (scenario 2 vs 1), 47% (scenario 3 vs 1), and 48% (scenario 2 vs 3) of iterations.

When plausible alternative values for critical parameters were used in the model, hospital cost savings and increases in QALYs were retained (Supplementary Table S9). When only simulations where a CRAB outbreak occurred were analyzed, the percentage of cost-effective iterations increased to 64% (Supplementary Table S10).

## Discussion

Our results showed the joint use of WGS and metagenomics were associated with smaller outbreaks, lower hospital costs, and an increase in accrued QALYs. Cost savings accrued from avoided treatments and fewer contact precautions resulting from fewer CRAB cases. We highlight the relatively small cost of WGS surveillance across 8 MDRO species (WGS cost AU$22,642 (US$15,397) and metagenomics cost AU$13,343 (US$9,073) and how they are dwarfed by microbiology culture costs (AU$1,099,269, US$747,503), antibiotic treatment costs (AU$125,622, US$85,423), and cost of PPE (AU$248,919, US$169,265). Sensitivity analyses indicated that WGS and metagenomics use was cost-effective in this context with a high degree of confidence when model inputs varied from their base values.

Several studies report on the economic benefits of WGS; however, to the best of our knowledge, this is the first economic evaluation on the use of metagenomics for infection control. Our previous work on an ESBL *E. coli* outbreak in 5 extended-stay wards predicted significant cost savings if WGS was implemented early^30^ and a budget impact analysis reported that statewide WGS surveillance targeting 6 MDROs could be cost saving.^31^ Furthermore, removing contact precautions for MDROs at low risk of spreading, confirmed from WGS data, may reduce hospital costs further.^14^ International studies have identified cost savings from WGS through interventions targeting transmission routes^15^ and reducing the spread of MRSA.^32^

Within our model, the stochastic processes created significant variance in the number of patients with CR-Ab across the scenarios that heavily drive the subsequent costs savings (−AU$781,840 to AU$264,347; −US$531,651 to US$179,756) and QALYs gained (−287 to 479). Approximately 20% of simulations resulted in no outbreaks occurring. Outbreak fadeouts are a hazard of stochastic outbreak simulations.^33^ When the ∼20% of iterations without an outbreak were removed, the likelihood of cost-effectiveness increased to 61% and 64% for scenario 2 and scenario 3 respectively (Supplementary Table S10). Understanding how and why stochastic simulations deviate from deterministic simulations is being viewed with increasing importance.^34^ For an outbreak to start, the pathogen must lie undetected long enough to spread to another patient or environmental object, which is influenced by stochastic parameters such as the patient’s length of stay, the regularity of screening, and ward transfer locations. The highly surveilled nature of the intensive care unit and burn unit added to the possibility that no outbreak occurs because patients would remain undetected for no longer than a few days. Several models are suitable for analyzing infection diseases. The trade-offs between compartmental models, agent-based simulation, and network-driven models are well documented.^35^ We chose an individual-based structure due to the data and computational software available to us, along with this level of detail being required to model the impact of WGS interventions and believe this is a valid choice.

The excessive cost and time to return a result has in the past been perceived as a limiting factor of implementing WGS into hospital practice.^8^ The cost of pathogen WGS continues to decrease, with international studies quoting the price between US$70,^15^ £100^32^ and AU$350 (US$238).^30^ The WGS cost of AU$150 (US$102) used in this study, the current operating price by our sequencing partners, consists of AU$124 (US$84) for the sequencing of the pathogen and AU$26 (US$18) for the bioinformatics. The bioinformatics costs dropped from AU$75 (US$51)^30^ due to the recently established pipeline creating a more automated analysis. Because WGS was not a dominant cost in the model, changing the cost of sequencing in sensitivity analysis did not change the outcomes. A 7-day WGS turnaround time is too long, although our model found little impact in reducing it to 3 days. This could be due to the outbreak occurring in already highly surveilled wards of the hospital. Reducing WGS turnaround time would likely have greater impact on outbreak size outside the ICU.^15^

This study had several limitations. This retrospective evaluation was based on a single hospital outbreak, which limits the generalizability of these findings. The results were dependent on decisions during model construction and several input parameters, which in line with the nature of infectious disease modeling, led to substantial uncertainty. Healthcare worker-to-patient transmission was not directly modeled, although spread to other rooms within the ward represents this type of transmission due to limited patient mobility. It was not possible to collect health utility scores directly from patients in this study, and scarce evidence was available in the literature. In the absence of other evidence, the sensitivity of metagenomics and microbiology cultures on detecting environmental CRAB contamination were estimated from 5 cases; however, sensitivity analyses on these values did not change the favorable incremental cost per QALY ratios. Parameters used in the model, like the environmental contamination values, did not have strong supporting evidence. Targeted metagenomics was used in this study because untargeted metagenomics as a surveillance tool has not been fully explored and would likely have lower sensitivity. Balanced against these limitations, is the use of a detailed simulation model, informed by accurate outbreak data, historical sequencing data, and 3 years of MDRO surveillance data. Our modeling incorporated the stochastic nature of outbreaks by limiting outbreak pathways through techniques like blocking, which removes unnecessary variation.^36^ Limiting the maximum number of transmissions to 3 in the binomial distribution formula was an example of blocking. On balance, based on our analysis and using realistic model parameters, targeted metagenomics, as used in this hospital, yielded good value for the money spent.

In conclusion, introducing WGS and metagenomics into infection control was likely to have favorable economic and clinical outcomes. The low proportion of costs attributed to sequencing all MDROs within the ICU and burns unit (<2%) highlights the manageable ongoing costs and encourages further sequencing studies in other hospital settings. Implementing these sequencing technologies is likely to yield decreased hospital costs, decreased infections, and increased QALYs.

## Supplementary material

For supplementary material accompanying this paper visit https://doi.org/10.1017/ash.2021.233. click here to view supplementary material
