# Supplementary material for: Cost-effectiveness analysis of whole-genome sequencing during an outbreak of carbapenem-resistant Acinetobacter baumannii
Source: Antimicrob Steward Healthc Epidemiol. 2021 Dec 13;1(1):e62. doi: 10.1017/ash.2021.233 (PMC9495627; doi:10.1017/ash.2021.233)
Supplement: Supplementary file 1 [file S2732494X21002333sup001.docx]

Supplementary Materials for manuscript ‘Cost-effectiveness of implementing genome sequencing during an outbreak of Carbapenem-resistant *Acinetobacter baumannii’*

Contents

[Outbreak description 1](#_Toc57887827)

[Scenario descriptions 2](#_Toc57887828)

[Detailed description of model structure 2](#_Toc57887829)

[Patient hospitalisation flow 2](#_Toc57887830)

[Pathogen transmission dynamics 3](#_Toc57887831)

[Infection Control Team 4](#_Toc57887832)

[Parameter estimation additional details 4](#_Toc57887833)

[ST1050 CRAB spread 4](#_Toc57887834)

[Environmental spread 5](#_Toc57887835)

[Non outbreak MRO Spread 5](#_Toc57887836)

[Ward admissions, ward transfers and ward stays 7](#_Toc57887837)

[Results 12](#_Toc57887838)

[References 15](#_Toc57887839)

# Outbreak description

The outbreak predominantly affected a 34 bed Intensive care unit (ICU) and an 18 bed Burns unit over a 32 month period. Extensive multi-drug resistant organism (MRO) surveillance was in place for these wards with microbiology screening occurring upon entry to the ICU, twice weekly in the ICU and weekly in Burns unit.

The outbreak was detected in the Burns unit of a 978-bed tertiary hospital in Queensland, Australia. In April of 2016 a patient with extensive burns was admitted to the ICU, initial nasal and rectal screening swabs were negative for multi-drug resistant pathogens, including Carbapenem-resistant *Acinetobacter baumannii* (CRAB). By day 45 of admission the patient had a positive blood culture for CRAB. Phenotypically similar CRAB was detected 18 times over the remainder of 2016, this included CRAB cases identified in ICU (6 cases) or other surgical wards throughout the hospital (2 cases), and eventually patients admitted to the Burns Unit (10 cases). A suspected outbreak of CRAB within the ICU instigated establishing an outbreak investigation team and characterizing the strain through whole genome sequencing (WGS). Figure 1 depicts the timing of infection control policy changes which impacted the CRAB outbreak. The reoccurring nature of the CRAB outbreak highly suggested that environmental transmission was involved. In 2016 and 2017, two rounds of extensive environment swabbing revealed no contamination in high-touch areas. Environmental metagenomics was introduced in November 2017, however not enough genetic content was collected for sequencing. In 2018, areas of high bacterial load, such as drains and burn baths, were targeted for environmental screening with metagenomics which revealed four areas positive for CRAB. Immediate reporting of WGS results were available when the CRAB outbreak resurfaced in May 2018.

# Scenario descriptions

We evaluated the different scenarios to investigate performance of different WGS availabilities and variations to pathogen’s transmission and infection potentials on the quality-adjusted life years (QALY), MRO cases, and total hospital cost. Microbiology culture and PCR were used to identify MROs and detection frequency rules were used to identify outbreaks (Figure 2).

Table S1: Sequencing implementation dates for each Scenario.

|  | Scenario 1 | Scenario 2 | Scenario 3 |
| --- | --- | --- | --- |
| WGS use, introduced | May of 2018 | Start of model (01/04/2016) | Start of model (01/04/2016) |
| WGS shotgun metagenomics use, introduced | November of 2017 | November of 2017 | Start of model (01/04/2016) |
| Swabbing of high bacterial load areas, introduced | July of 2018 | Start of model (01/04/2016) | Start of model (01/04/2016) |

Abbreviations: WGS – whole genome sequencing

# Detailed description of model structure

The simulation model developed in the AnyLogic software combines elements of population-level dynamics using discrete event simulation and individual-level dynamics using agent-based modelling in order to simulate complex, interacting processes typical of a hospital outbreak. There are three main interacting components of this hybrid simulation model:

1. Patient hospitalisation flow dynamics
2. Pathogen transmission dynamics
3. Outbreak management actions

which are elaborated further in the following subsections. In addition to allowing interaction between processes on different scales, AnyLogic allows the processes to interact with the spatial location of the “agents” which are chiefly patients, but could also be pathogens or inanimate objects such as patient beds. This feature is useful for structured contacts typical of hospital interactions, e.g., patients could interact with other patients in the same bed bay except those in isolation rooms in the ward.

## Patient hospitalisation flow

Patient hospitalisation flow dynamics were modelled at an individual patient level. A patient is admitted to one of six bed groupings. Beds within the model are grouped into multi-bed room ICU, single-bed room ICU, multi-bed room Burns unit, single-bed room Burns unit, infectious disease ward and all other hospital wards grouped together. Admitted patients could be transferred to another bed location, discharged or discharged awaiting readmission. Patient movement through the hospital is informed through admission probability, transfer probability and ward length of stay estimates obtained using the Hospital Based Corporate Information System (HBCIS) dataset.

## Pathogen transmission dynamics

Pathogen transmission dynamics for the ST1050 CRAB outbreak were modelled at the ward level through patient-to-patient transmission and contaminated room to patient transmission. The patient to patient transmission was modelled assuming that new colonisations in a ward are a daily function of the number of patients in the ward who are currently colonised/infected with ST1050 CRAB using a binomial distribution formula ( ${}_{N}{C{}_{X}}* P^{X}{(1-P)}^{(n-x)}$ ), where x is the number of transmissions (limited to 3), n is the number of susceptible patients and P is the probability. The daily probability was calculated using $1-\exp\left\{ \frac{-\beta C}{S+C} \right\}$, where the exponent is derived from the frequency-dependent transmission term [1], with transmission parameter$(\beta)$,number of susceptible patients$(S)$, and number of colonized/infected patients($C)$. The number of daily transmissions were limited to three due to the variation created in the stochastic model when this was relaxed. The discrete-time event model calculated new transmissions daily. The new transmissions do not impact the susceptible patient probability until the next day. Once the number of new colonised patients was calculated, susceptible patients were selected weighted by their proximity to a colonised/infected patient. Patient proximity is measured via the distance in the hospital network environment. We assumed that colonized patients remained colonized for the rest of their hospitalisation. Healthcare worker (HCW) to patient transmission was not directly modelled due to data limitations. As patients in the ICU and Burns unit generally have limited mobility, there is an implicit assumption that transmission is facilitated by transiently contaminated healthcare workers or shared hospital equipment [2]. As patient movement within the ward does not occur, spread to other rooms within the ward represents HCW – patient transmission. Due the stark differences in transmission throughout the outbreak, the patient-to-patient β value is reduce on 1/10/2016. The contaminated room to patient transmission used the same formula, where $\beta$ is the environmental transmission parameter, $S$is 1 if the patient in the bed is susceptible, $C$ is the number of colonised objects around the bed, and $N$ is the number of objects and susceptible patients in the bed. Each of high touch areas (HTA) and aqueous reservoir being contaminated accounts for one colonised object.

Other MROs were present in the intensive care and burn unit throughout the outbreak. These MROs consisted of methicillin resistant *Staphylococcus aureus* (MRSA), extended spectrum β-lactamase producing *Escherichia coli* (ESBL *E. coli*), vancomycin-resistant *Enterococcus faecium* (VRE), ESBL-producing *Klebisella pneumoniae* (ESBL K. *pneumoniae*), carbapenemase-producing Enterobacterales (CPE), CRAB, *Pseudomonas aeruginosa* and *Clostridioides* *difficile* infection (CDI). MROs were detected based on incident daily probabilities. Clusters were identified for ESBL K. *pneumoniae* and ESBL *E. coli*. These MROs continue to spread at a specific daily probability until the predetermined cluster size was reached.

At any point in time in the model, a patient can be classified as either susceptible, colonised or infected. A hospital bed can be classified as clean, high-touch area (HTA) contaminated, aqueous reservoir contaminated or both HTA and aqueous reservoir contaminated. Each instance an infected/colonised patient enters a room, there is a probability of contaminating either the HTA or aqueous reservoir.

## Infection Control Team

The intensive care and burns unit have strict screening protocols in place to prevent unknown spread of dangerous pathogens around vulnerable patients. The ICU is screened twice weekly and the Burns unit is screen once each week. Microbiological screening is assumed to take 2-days to process, in line with current practice at the hospital. Positive microbiological cultures trigger contact precautions, which consist of contract tracing, isolating the patient, use of personal protective equipment (PPE) by staff and environmental cleaning of patient’s beds. A phenotypically confirmed outbreak was triggered when three CRAB samples were detected within five days, which enacted ward-wide screening. If a CRAB pathogen is detected within the following week, then environmental screening is instigated. Positive cultures result in improved cleaning of environmental contaminated beds. The outbreak is declared ceased after one week of no detections. When WGS was available, a positive microbiological culture was sequenced, which took seven days to process. An outbreak was declared when two sequenced CRAB cases were identified as the same strain and re-declared after each additional linked case. When Metagenomics was available, environmental swabs were sent for sequencing which took seven days to process

Patient events modelled consume healthcare resources in the form of staff time (e.g., nurses, cleaning staff, lab technicians) and consumables (microbiology culture set, cleaning products). Costs estimates are attached to these events and are accumulated over the simulation period to derive the cost outcomes for the evaluation.

# Parameter estimation additional details

## ST1050 CRAB spread

**Transmission parameter calibration**

The transmission parameter, β, governs how readily the outbreak spreads. As the outbreak spreads quickly at the start before tapering off, transmission is determined by four β parameters. These are the initial patient-patient transmission parameter, initial environment-patient transmission parameter, 2^nd^ patient-patient transmission parameter and 2^nd^ environment-patient transmission parameter. The four transmission parameter estimates and the probability of contaminating an aqueous reservoir presented in the main text were calibrated over 50,000 iterations, to generate simulations matching the observed outbreak at key time points. These were 11 time points over three years matching the end of each quarter. Each iteration was run 100 times to average the outcomes for those parameter groupings. This is due to the variation in outbreak pathways.

Figure S1: Observed vs Calibrated Detected Colonizations


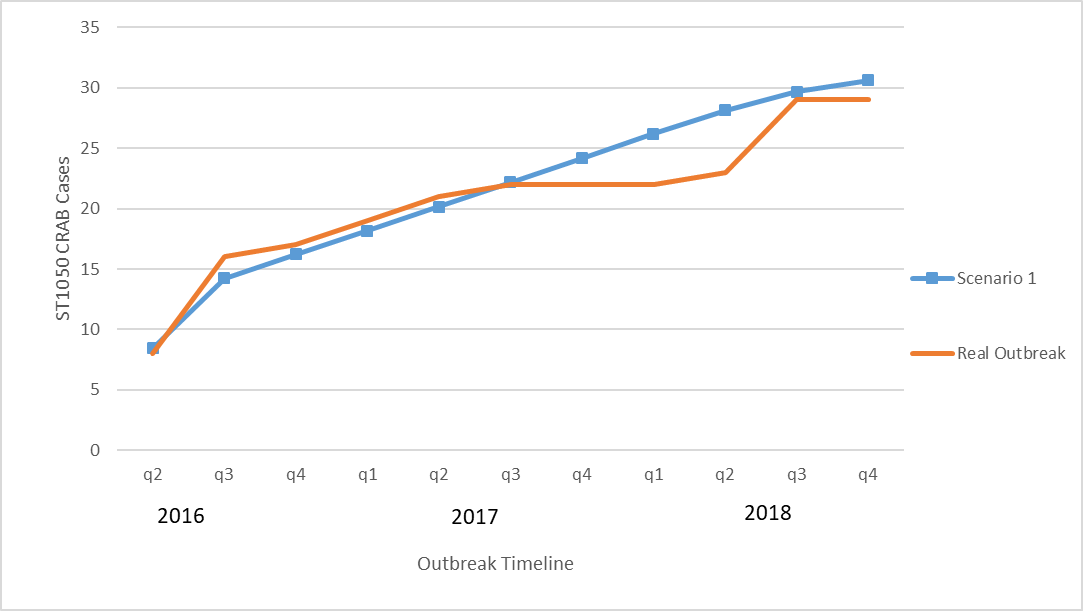


Abbreviations: CRAB – *Carbapenem-resistant Acinetobacter baumannii*; q – quarter;

## Environmental spread

The probability of HTA and/or aqueous reservoirs becoming contaminated incorporates the likelihood that future cleaning of the room will fail. The CRAB environmental contamination was sourced from a one-day point prevalence screening and one-month surveillance of clinical cultures for CRAB. Five rooms were found to be contaminated with CRAB from eight rooms with a CRAB Patient. This led to a CRAB environmental contamination likelihood of 0.63. The environmental cleaning success rate was sourced from a CRAB cleaning evaluation study. Basic environmental cleaning had a success rate of 0.78 for single-bed rooms and 0.85 for multi-bed rooms. These parameters were combined and the probability of a single-bed room being contaminated of 0.14 and multi-bed room of 0.09 (Table 1). These studies were the best published estimates available, although due to the low sample sizes and observational design were low quality-of-evidence.

Undetected environmental contamination is likely the primary cause of the CRAB outbreak [3]. The environmental contamination likely remain undetected due to the low sensitively of microbiology cultures. Based on the outbreak data microbiology cultures detected environmental CRAB in 40% of positive locations. Metagenomics detected CRAB in 80% of positive locations.

## Non outbreak MRO Spread

The spread of other MROs were calculated from three years of MRO surveillance data from within the intensive care and burns units. A daily detection probability was calculated for each of MRSA, ESBL E. coli, VRE, ESBL K. *pneumoniae*, CPE, CRAB, *Pseudomonas aeruginosa* and CDI for each of 2015, 2016 and 2017 (Table S2). Clusters within these MROs were identified for ESBL K. *pneumoniae* and ESBL *E. coli*, based on two years (December 2017 to December 2019) of MRO sequencing data. Genetic relatedness was determined by examining the number of core genome single nucleotide polymorphisms (SNP) that differ between any two isolates (pair-wise core genome SNP distance). An assumption was made that MRO clusters detected with WGS were limited to three cases. The bloodstream, respiratory and urinary tract infection rates for each of these pathogens were estimated based on corresponding ICD-10 codes (Table S2). The frequency of deaths in hospital from patients infected with any of the MROs were obtained from published reports and ranged from 0.7% for CDI to 36.6% for VRE (Table S2). Environmental transmission was not modelled for these pathogens due to data limitations and modelling simplification. The non-outbreak MRO spread parameters were estimated from large data sets and were accurately measured. The quality-of-evidence of these parameters did not contribute to outcome uncertainty.

Table S2: Non-outbreak multi-resistant organism incidence, mortality and LOS

| Pathogen | Incidence rate -  2016 | Incidence rate -2017 | Incidence rate -  2018 | Source | Mortality rate | Source | LOS post detection | Source |
| --- | --- | --- | --- | --- | --- | --- | --- | --- |
| Non-ST1050 CR-Ab | 0.013 | 0.002 | 0.002 | RBWH Surveillance data | 7.7% | AGAR, 2018 | 11 Days | A´ lvarez-Marı´n 2016 |
| ESBL E. coli | 0.045 | 0.054 | 0.034 |  | 14.4% |  | 17 Days | Suzuki 2020 |
| MRSA | 0.016 | 0.012 | 0.047 |  | 17.7% |  | 35 Days | Kirwin. 2019 |
| ESBL KP. | 0.003 | 0.012 | 0.002 |  | 20.0% |  | 17 Days | Suzuki 2020 |
| PA | 0.023 | 0.010 | 0.002 |  | 28.6% |  | 9 Days | Kaier 2019 |
| CPE | 0.003 | 0.000 | 0.010 |  | 6.7% |  | 15 Days | Rodriguez-Acevedo . 2020 |
| VRE | 0.019 | 0.017 | 0.020 |  | 36.6% |  | 14 Days | Lloyd-smith (2013) |
| CDI | 0.013 | 0.012 | 0.017 |  | 0.7% | ACSQHC 2018 | 5 Days | Lagu 2014 |

Abbreviations: MRSA – Methicillin-resistant *staphylococcus aureus*, ESBL - Extended spectrum beta-lactamases, E. coli - *Escherichia coli*; KP - *Klebsiella pneumoniae*; VRE - Vancomycin-resistant enterococci, CPE - Carbapenemase-Producing Enterobacterales , CRAB - Carbapenem-resistant *Acinetobacter baumannii*, PA - *Pseudomonas aeruginosa*; CDI - *Clostridioides difficile* infection;

Table S3: Multi-resistant organism infection rate and cost

| Infections | Rate/Cost | Source | Infections | Rate/Cost | Sources |
| --- | --- | --- | --- | --- | --- |
| CRAB - Sepsis rate | 0.77 | ICD-10 (OGN)(S) | PA - Sepsis rate | 0.57 | ICD-10 (PA)(S) |
| - RTI rate | 0.50 | ICD-10 (OGN)(R) | - RTI rate | 0.29 | ICD-10 (PA)(R) |
| - UTI rate | 0.27 | ICD-10 (OGN)(U) | - UTI rate | 0.33 | ICD-10 (PA)(U) |
| - Colistin + tigecycline ^a^ or Colistin + Meropenem ^b^ | $3,199 | Viehman (2014) [4] and Hospital Pharmacy pricing | - piperacillin-tazobactam + Colistin ^g^ | $2,191 | kwee (2015) [5] |
| ESBL E. coli - Sepsis rate | 0.83 | ICD-10 (Ec)(S) | CPE - Sepsis rate | 1.00 | ICD-10 (GN)(S) |
| - RTI rate | 0.25 | ICD-10 (Ec)(R) | - RTI rate | 0.00 | ICD-10 (GN)(R) |
| - UTI rate | 0.58 | ICD-10 (Ec)(U) | - UTI rate | 1.00 | ICD-10 (GN)(U) |
| - Meropenem ^d^ | $321 | Wozniak (2018)[6] and Hospital | - Colistin + Meropenem ^b^ or Gentamicin/Amikacin ^f^ | $2,385 / $3,041 | Pharmacy infection network [7] and Hospital Pharmacy pricing |
| MRSA - Sepsis rate | 0.68 | ICD-10 (SA)(S) | VRE - Sepsis rate | 0.77 | ICD-10 (O)(S) |
| - RTI rate | 0.28 | ICD-10 (SA)(R) | - RTI rate | 0.15 | ICD-10 (O)(R) |
| - UTI rate | 0.30 | ICD-10 (SA)(U) | - UTI rate | 0.38 | ICD-10 (O)(U) |
| - Flucloxacillin & Vancomycin ^c^ | $580 | SA guideline [8] / Hospital Pharm | - Linezolid or Daptomycin ^e^ | $1,786 / $4,585 | Pharmacy pricing |
| ESBL KP. - Sepsis rate | 0.72 | ICD-10 (KP)(S) | CDI - Sepsis rate | 1.00 | ICD-10 (C)(S) |
| - RTI rate | 0.16 | ICD-10 (KP)(R) | - RTI rate | 0.00 | ICD-10 (C)(R) |
| - UTI rate | 0.41 | ICD-10 (KP)(U) | - UTI rate | 1.00 | ICD-10 (C)(U) |
| - Meropenem ^d^ | $321 | Wozniak (2018)[6] and Hospital | - Vancomycin ^h^ | $176 | Al-Jashaami (2016) [9] |

Abbreviations: MRSA – Methicillin-resistant *staphylococcus aureus*, ESBL - Extended spectrum beta-lactamases*, E. coli* - *Escherichia coli*; KP - *Klebsiella pneumoniae*; VRE - Vancomycin-resistant *Enterococci*, CPE - Carbapenemase-Producing *Enterobacterales* , CRAB - Carbapenem-resistant *Acinetobacter baumannii*, PA - *Pseudomonas aeruginosa*; CDI - *Clostridioides* *difficile* infection; UTI - urinary tract infection; RTI - respiratory tract infection;

ICD-10 item legend: (S) = Sepsis: A41.0, A41.1, A41.2, A41.50, A41.51, A41.52, A41.58, A41.8, A41.9; (R) = RTI: J15.0, J15.1, J15.2, J15.5, J15.6, J15.7, J15.8, J15.9, J17.0; (U) = UTI: N39.0; (OGN) = gram-negative organism: A41.58, J15.6; (Ec) = E. Coli: J15.5, A41.51, A04.1, A04.2, A04.4; (SA) = *Staphylococcus aureus*: A41.0, A41.1, A41.2, A49.01, B95.7, J15.2; (KP) = *Klebsiella pneumonia*: B96.1, J15.0; (PA) = *Pseudomonas aeruginosa*: B96.5, J15.1, A41.52; (GN) = unspecified gram-negative: A41.50; (O) = Other: A41.8, A49.8, B94.8, J15.8, A04.8; (C) = CDI: A04.7. All infections required resistance code of Z06.51, Z06.52, Z06.53, Z06.58, Z06.60, Z06.61, Z06.62, Z06.63, Z06.67, Z06.69, Z06.70, Z06.77, Z06.78.

^a^ Colistin administered at 275mg for 14 days and tigecycline administered at 100mg followed by 50mg every 12 hours for 14 days

^b^ Colistin administered at 275mg for 14 days and Meropenem administered at 1.0-2g for 3 times daily for 14 days

^c^  Flucloxacillin administered at 2g IV 6 hourly for 2 days and Vancomycin at 2g for 21 days

^d^ Meropenem administered at 1.0-2g for 3 times daily for 14 days

^e^ Linezolid administered at 2×0.6 g for 14 days and Daptomycin 0.6g daily for 14 days

^f^ Gentamicin administered at 5-7mg/kg for 10 days and Amikacin administered at 15mg/kg for 10 days

^g^ Colistin administered at 275mg for 14 days and tazobactam administered at 4.5g every 6 hours for 8 days

^h^ Vancomycin at 2g for 21 days

## Ward admissions, ward transfers and ward stays

Patient hospitalisations were represented in the model as a series of ward transfers, with each ward stay duration dependent on the current ward as well as where patients are moved to subsequently (including readmission and discharge from the hospital). Ward stays were estimated as independent Gamma distributions for all observed ward pair combinations in the data set using the methods of moments [10]. Hospital daily admission rate, ward admissions, ward transfers proportions and ward length of stays were estimated empirically from the HBICIS data set.

In the simulation runs, patients were initialised in to the wards based on the distribution of patients who were in the hospital at the start of the HBCIS data set (1^st^ April 2016) (“Existing patients” column in Table S4). New admissions into the hospital wards during the study period were assigned into one of the 6 study groupings based on the proportions listed in the “New admissions” columns of Table S4. Estimates of first ward transfer proportions and ward stay durations (Table S5) and future ward transfers (Table S6) were derived for new admissions during the study period. Specific ward transfers and length of stays were derived for undetected patients with CRAB (Table S7) and Detected patients with CRAB (Table S8).

Table S4: Distribution of existing patients’ ward at start of study period (existing patients) and first ward for new admissions (new admissions).

|  | Percentage of admissions (%) | | | |
| --- | --- | --- | --- | --- |
| Ward | Existing patients | New admissions 2016 | New admissions 2017 | New admissions 2018 |
| ICU G | 15% | 43% | 42% | 44% |
| ICU S | 5% | 3% | 4% | 3% |
| Burn G | 7% | 10% | 13% | 11% |
| Burn S | 8% | 5% | 7% | 7% |
| Other | 62% | 39% | 35% | 34% |
| ID | 3% | 0% | 0% | 0% |

Abbreviations: ICU – intensive care unit; G – group room; S – single bed room; other – all other hospital wards excluding the 3 study wards; ID – infectious disease

Table S5: Number, proportion and ward stay estimates of the different ward pair combinations for patients after admission

|  | | 2016 | | | | | 2017 | | | | | 2018 | | | | |
| --- | --- | --- | --- | --- | --- | --- | --- | --- | --- | --- | --- | --- | --- | --- | --- | --- |
| Ward transfer pairing | | No. of tran. | Tran. Prob. | LOS | Gamma distribution estimates | | No. of tran. | Tran. pro. | LOS | Gamma distribution estimates | | No. of tran. | Tran. pro. | LOS | Gamma distribution estimates | |
| from | to |  |  |  | shape | scale |  |  |  | shape | scale |  |  |  | shape | scale |
| ICU G | ID | 5 | 0.003 | 2.000 | 4.000 | 0.500 | 7 | 0.003 | 2.571 | 2.525 | 1.019 | 7 | 0.002 | 9.714 | 0.516 | 18.828 |
| ICU G | Dis | 47 | 0.026 | 5.255 | 1.347 | 3.901 | 75 | 0.027 | 5.400 | 2.180 | 2.477 | 69 | 0.021 | 5.971 | 0.550 | 10.861 |
| ICU G | ICU S | 11 | 0.006 | 8.000 | 0.448 | 17.850 | 22 | 0.008 | 4.636 | 0.397 | 11.679 | 14 | 0.004 | 4.143 | 0.278 | 14.886 |
| ICU G | Burn S | 6 | 0.003 | 4.000 | 0.909 | 4.400 | 9 | 0.003 | 4.333 | 1.138 | 3.808 | 12 | 0.004 | 6.417 | 1.113 | 5.765 |
| ICU G | Other | 702 | 0.390 | 3.735 | 0.090 | 41.272 | 1059 | 0.379 | 3.046 | 0.531 | 5.741 | 1370 | 0.411 | 3.047 | 0.439 | 6.938 |
| ID | ICU S | 4 | 0.002 | 6.000 | 0.360 | 16.667 |  |  |  |  |  |  |  |  |  |  |
| ICU S | ICU G | 7 | 0.004 | 7.286 | 0.620 | 11.745 | 19 | 0.007 | 6.526 | 1.601 | 4.075 | 11 | 0.003 | 7.182 | 0.679 | 10.577 |
| ICU S | ID | 3 | 0.002 | 11.667 | 1.398 | 8.343 | 12 | 0.004 | 6.583 | 1.220 | 5.398 | 5 | 0.001 | 6.600 | 0.502 | 13.152 |
| ICU S | Dis | 6 | 0.003 | 13.167 | 2.313 | 5.694 | 8 | 0.003 | 10.625 | 1.741 | 6.103 | 9 | 0.003 | 4.889 | 1.081 | 4.523 |
| ICU S | Burn S | 12 | 0.007 | 18.083 | 0.935 | 19.339 | 21 | 0.008 | 10.619 | 1.259 | 8.433 | 23 | 0.007 | 10.304 | 1.156 | 8.914 |
| ICU S | Other | 30 | 0.017 | 4.833 | 0.915 | 5.281 | 40 | 0.014 | 3.450 | 1.538 | 2.244 | 57 | 0.017 | 3.123 | 0.683 | 4.575 |
| Burn G | ICU G | 6 | 0.003 | 2.000 | 3.333 | 0.600 | 10 | 0.004 | 1.700 | 1.616 | 1.052 | 11 | 0.003 | 1.727 | 1.844 | 0.937 |
| Burn G | ReAd | 15 | 0.008 | 5.400 | 1.188 | 4.545 | 19 | 0.007 | 7.105 | 0.422 | 16.856 | 13 | 0.004 | 10.769 | 1.855 | 5.806 |
| Burn G | Dis | 120 | 0.067 | 6.658 | 1.816 | 3.666 | 259 | 0.093 | 5.282 | 1.806 | 2.924 | 290 | 0.087 | 5.810 | 1.655 | 3.510 |
| Burn G | Burn S | 14 | 0.008 | 3.286 | 0.530 | 6.201 | 23 | 0.008 | 1.348 | 1.468 | 0.918 | 28 | 0.008 | 1.964 | 0.876 | 2.243 |
| Burn G | Other | 19 | 0.011 | 4.000 | 0.993 | 4.028 | 46 | 0.016 | 4.130 | 0.702 | 5.882 | 40 | 0.012 | 2.800 | 1.573 | 1.780 |
| Burn S | ICU G | 3 | 0.002 | 1.000 | 0.000 | 0.000 | 3 | 0.001 | 1.333 | 5.333 | 0.250 | 3 | 0.001 | 3.667 | 0.938 | 3.909 |
| Burn S | ReAd | 8 | 0.004 | 5.875 | 4.569 | 1.286 | 13 | 0.005 | 20.538 | 0.175 | 117.273 | 16 | 0.005 | 5.625 | 1.193 | 4.714 |
| Burn S | Dis | 51 | 0.028 | 8.824 | 1.078 | 8.181 | 126 | 0.045 | 6.849 | 1.546 | 4.429 | 170 | 0.051 | 7.294 | 1.290 | 5.655 |
| Burn S | Burn G | 19 | 0.011 | 6.316 | 0.467 | 13.512 | 31 | 0.011 | 6.290 | 1.075 | 5.852 | 35 | 0.010 | 4.086 | 0.971 | 4.209 |
| Burn S | Other | 5 | 0.003 | 5.400 | 0.480 | 11.259 | 22 | 0.008 | 3.500 | 1.500 | 2.333 | 21 | 0.006 | 5.571 | 1.254 | 4.444 |
| Other | ICU G | 452 | 0.251 | 4.657 | 0.407 | 11.454 | 604 | 0.216 | 4.561 | 0.193 | 23.600 | 729 | 0.218 | 4.056 | 0.444 | 9.138 |
| Other | ID | 9 | 0.005 | 10.556 | 0.855 | 12.342 | 6 | 0.002 | 3.667 | 1.310 | 2.800 | 13 | 0.004 | 1.231 | 4.220 | 0.292 |
| Other | ReAd | 21 | 0.012 | 9.000 | 1.171 | 7.689 | 15 | 0.005 | 12.267 | 0.402 | 30.530 | 7 | 0.002 | 14.000 | 5.297 | 2.643 |
| Other | Dis | 117 | 0.065 | 12.624 | 1.297 | 9.732 | 144 | 0.052 | 10.201 | 1.208 | 8.442 | 156 | 0.047 | 11.282 | 1.505 | 7.498 |
| Other | ICU S | 28 | 0.016 | 4.964 | 0.540 | 9.199 | 60 | 0.021 | 7.250 | 0.406 | 17.854 | 81 | 0.024 | 7.543 | 0.978 | 7.712 |
| Other | Burn G | 57 | 0.032 | 1.930 | 0.976 | 1.978 | 96 | 0.034 | 2.823 | 0.837 | 3.371 | 87 | 0.026 | 2.471 | 0.451 | 5.475 |
| Other | Burn S | 22 | 0.012 | 2.864 | 0.593 | 4.832 | 42 | 0.015 | 4.833 | 0.138 | 34.929 | 52 | 0.016 | 2.558 | 0.381 | 6.707 |
| ID | ICU G |  |  |  |  |  |  |  |  |  |  | 5 | 0.001 | 15.800 | 0.615 | 25.709 |
| ID | Other |  |  |  |  |  |  |  |  |  |  | 3 | 0.001 | 7.667 | 1.191 | 6.435 |

Abbreviations: ICU – intensive care unit; G – group room; S – single bed room; other – all other hospital wards excluding the 3 study wards; ID – infectious disease; ReAd – readmission; Dis – discharge; tran. – transfer; prob. – Probability; LOS – length of stay;

Table S6: Number, proportion and ward stay estimates of the different ward pair combinations for patients after 1^st^ transfer

|  | | 2016 | | | | | 2017 | | | | | 2018 | | | | |
| --- | --- | --- | --- | --- | --- | --- | --- | --- | --- | --- | --- | --- | --- | --- | --- | --- |
| Ward transfer pairing | | No. of tran. | Tran. pro | LOS | Gamma distribution estimates | | No. of tran. | Tran. pro  shape | LOS | Gamma distribution estimates | | No. of tran. | Tran. pro. | LOS | Gamma distribution estimates | |
| from | to |  |  |  | shape | scale |  |  |  | shape | scale |  |  |  | shape | scale |
| ICU G | ID | 3 | 0.003 | 8.33 | 0.43 | 19.36 | 5 | 0.003 | 1.40 | 6.53 | 0.21 | 5 | 0.003 | 2.80 | 3.56 | 0.79 |
| ICU G | Dis | 12 | 0.012 | 6.00 | 1.40 | 4.27 | 7 | 0.005 | 6.14 | 1.07 | 5.72 | 11 | 0.006 | 3.73 | 2.47 | 1.51 |
| ICU G | ICU S | 3 | 0.003 | 7.00 | 2.58 | 2.71 | 3 | 0.002 | 9.67 | 0.78 | 12.45 | 8 | 0.005 | 2.25 | 1.06 | 2.13 |
| ICU G | Burn S | 4 | 0.004 | 2.50 | 1.70 | 1.47 | 7 | 0.005 | 2.00 | 4.00 | 0.50 | 3 | 0.002 | 1.33 | 5.33 | 0.25 |
| ICU G | Other | 105 | 0.103 | 3.24 | 0.50 | 6.52 | 156 | 0.105 | 2.83 | 0.97 | 2.92 | 190 | 0.112 | 2.90 | 0.66 | 4.40 |
| ID | ReAd | 4 | 0.004 | 7.50 | 1.37 | 5.47 | 3 | 0.002 | 14.67 | 1.88 | 7.80 |  |  |  |  |  |
| ID | Dis | 27 | 0.027 | 10.78 | 0.82 | 13.09 | 28 | 0.019 | 10.93 | 0.70 | 15.62 | 28 | 0.017 | 11.75 | 1.61 | 7.31 |
| ReAd | ICU G | 66 | 0.065 | 323.03 | 1.55 | 207.83 | 96 | 0.064 | 203.48 | 1.41 | 143.84 | 48 | 0.028 | 109.56 | 1.29 | 84.88 |
| ReAd | ICU S | 5 | 0.005 | 117.60 | 0.58 | 201.59 | 5 | 0.003 | 201.80 | 0.46 | 435.67 |  |  |  |  |  |
| ReAd | Burn G | 15 | 0.015 | 243.33 | 1.05 | 231.91 | 24 | 0.016 | 128.67 | 1.33 | 96.97 | 24 | 0.014 | 83.17 | 0.68 | 122.41 |
| ReAd | Burn S | 17 | 0.017 | 236.47 | 0.95 | 248.26 | 18 | 0.012 | 125.44 | 0.82 | 153.82 | 11 | 0.006 | 70.00 | 0.51 | 136.19 |
| ReAd | Other | 69 | 0.068 | 250.22 | 0.95 | 263.36 | 88 | 0.059 | 198.70 | 1.14 | 174.87 | 54 | 0.032 | 101.56 | 1.15 | 88.42 |
| ICU S | ID | 3 | 0.003 | 2.00 | 4.00 | 0.50 | 3 | 0.002 | 10.67 | 1.74 | 6.13 | 4 | 0.002 | 5.25 | 2.03 | 2.59 |
| ICU S | Dis | 5 | 0.005 | 13.60 | 0.91 | 14.88 | 7 | 0.005 | 10.29 | 0.86 | 11.98 | 9 | 0.005 | 5.33 | 1.08 | 4.92 |
| ICU S | Other | 10 | 0.010 | 2.50 | 3.41 | 0.73 | 25 | 0.017 | 3.88 | 0.64 | 6.06 | 47 | 0.028 | 4.87 | 0.65 | 7.50 |
| Burn G | Dis | 12 | 0.012 | 2.83 | 2.48 | 1.14 | 24 | 0.016 | 6.42 | 1.62 | 3.97 | 28 | 0.017 | 4.71 | 1.33 | 3.55 |
| Burn S | ICU G | 3 | 0.003 | 8.67 | 2.48 | 3.50 |  |  |  |  |  |  |  |  |  |  |
| Burn S | ReAd | 7 | 0.007 | 34.43 | 2.09 | 16.46 | 8 | 0.005 | 39.50 | 0.65 | 60.90 | 10 | 0.006 | 23.80 | 0.98 | 24.32 |
| Burn S | Dis | 9 | 0.009 | 22.11 | 4.00 | 5.52 | 12 | 0.008 | 26.75 | 1.56 | 17.18 | 26 | 0.015 | 25.19 | 0.23 | 107.46 |
| Burn S | Burn G | 3 | 0.003 | 12.67 | 13.01 | 0.97 | 4 | 0.003 | 13.50 | 0.85 | 15.88 |  |  |  |  |  |
| Other | ICU G | 42 | 0.041 | 9.40 | 0.65 | 14.43 | 55 | 0.037 | 10.38 | 0.50 | 20.81 | 51 | 0.030 | 7.84 | 0.58 | 13.48 |
| Other | ID | 15 | 0.015 | 11.27 | 1.49 | 7.54 | 9 | 0.006 | 8.89 | 0.80 | 11.07 | 6 | 0.004 | 6.17 | 0.87 | 7.10 |
| Other | ReAd | 80 | 0.079 | 17.95 | 0.28 | 64.98 | 103 | 0.069 | 10.77 | 0.36 | 29.58 | 46 | 0.027 | 17.24 | 0.19 | 91.91 |
| Other | Dis | 494 | 0.486 | 10.03 | 1.16 | 8.65 | 779 | 0.523 | 9.25 | 0.67 | 13.78 | 1059 | 0.624 | 10.32 | 0.61 | 17.02 |
| Other | ICU S | 3 | 0.003 | 4.33 | 4.33 | 1.00 | 6 | 0.004 | 20.33 | 2.45 | 8.30 | 23 | 0.014 | 9.83 | 0.76 | 13.00 |
| ID | ICU G |  |  |  |  |  | 3 | 0.002 | 8.67 | 1.13 | 7.65 |  |  |  |  |  |
| ID | Other |  |  |  |  |  | 7 | 0.005 | 5.14 | 1.27 | 4.05 | 5 | 0.003 | 13.40 | 0.44 | 30.21 |
| ReAd | ID |  |  |  |  |  | 5 | 0.003 | 84.20 | 0.45 | 187.17 |  |  |  |  |  |
| ICU S | ICU G |  |  |  |  |  | 4 | 0.003 | 4.00 | 1.85 | 2.17 |  |  |  |  |  |
| Burn G | Other |  |  |  |  |  | 5 | 0.003 | 2.00 | 2.00 | 1.00 | 6 | 0.004 | 5.00 | 1.14 | 4.40 |
| Burn S | Other |  |  |  |  |  | 4 | 0.003 | 35.50 | 1.45 | 24.50 | 4 | 0.002 | 6.25 | 0.40 | 15.51 |
| Other | Burn S |  |  |  |  |  | 4 | 0.003 | 6.50 | 0.52 | 12.56 | 3 | 0.002 | 6.33 | 0.87 | 7.32 |
| ICU S | Burn S |  |  |  |  |  |  |  |  |  |  | 4 | 0.002 | 5.25 | 0.38 | 13.76 |
| Burn G | Burn S |  |  |  |  |  |  |  |  |  |  | 3 | 0.002 | 4.33 | 0.77 | 5.62 |
| Other | Burn G |  |  |  |  |  |  |  |  |  |  | 3 | 0.002 | 17.67 | 0.48 | 36.58 |

Abbreviations: ICU – intensive care unit; G – group room; S – single bed room; other – all other hospital wards excluding the 3 study wards; ID – infectious disease; ReAd – readmission; Dis – discharge; tran. – transfer; prob. – Probability; LOS – length of stay;

Table S7: Number, proportion and ward stay estimates of the different ward pair combinations for patients once colonised with ST1050 CRAB

|  | | Colonised patient movement | | | | |
| --- | --- | --- | --- | --- | --- | --- |
| Ward transfer pairing | | No. of tran. | Tran. pro | LOS | Gamma distribution estimates | |
| from | to |  |  |  | shape | scale |
| ICU G | ICU S | 2 | 0.024 | 9.00 | 4.50 | 2.00 |
| ICU G | Burn S | 2 | 0.024 | 4.00 | 8.00 | 0.50 |
| ICU G | Other | 3 | 0.037 | 8.67 | 2.85 | 3.04 |
| ICU S | ICU G | 3 | 0.037 | 2.67 | 21.33 | 0.13 |
| ICU S | ID | 1 | 0.012 | 45.00 | 45.00 | 0.00 |
| ICU S | Dis | 1 | 0.012 | 19.00 | 19.00 | 0.00 |
| ICU S | Burn S | 15 | 0.183 | 23.80 | 1.29 | 18.45 |
| Burn G | ReAd | 2 | 0.024 | 2.00 | 2.00 | 0.00 |
| Burn G | Dis | 6 | 0.073 | 13.67 | 4.30 | 3.18 |
| Burn G | Burn S | 3 | 0.037 | 9.67 | 7.58 | 1.28 |
| Burn S | ICU G | 1 | 0.012 | 14.00 | 14.00 | 0.00 |
| Burn S | ReAd | 13 | 0.159 | 33.08 | 0.43 | 76.48 |
| Burn S | Dis | 18 | 0.220 | 26.33 | 0.81 | 32.55 |
| Burn S | ICU S | 2 | 0.024 | 25.50 | 0.59 | 43.31 |
| Burn S | Burn G | 4 | 0.049 | 25.00 | 0.83 | 30.00 |
| Burn S | Burn S | 1 | 0.012 | 124.00 | 124.00 | 0.00 |
| Burn S | Other | 5 | 0.061 | 58.20 | 1.95 | 29.79 |

Abbreviations: ICU – intensive care unit; G – group room; S – single bed room; other – all other hospital wards excluding the 3 study wards; ID – infectious disease; ReAd – readmission; Dis – discharge; tran. – transfer; prob. – Probability; LOS – length of stay;

Table S8: Number and ward stay estimates of the different ward pair combinations for patients once detected with ST1050 CRAB

|  | | 2016 | | | | 2017 | | | | 2018 | | | |
| --- | --- | --- | --- | --- | --- | --- | --- | --- | --- | --- | --- | --- | --- |
| Ward transfer pairing | | No. of tran. | LOS | Gamma distribution estimates | | No. of tran. | LOS | Gamma distribution estimates | | No. of tran. | LOS | Gamma distribution estimates | |
| from | to |  |  | shape | scale |  |  | shape | scale |  |  | shape | scale |
| ICU S | Dis | 3.00 | 41 | 1.29 | 31.90 | 8.00 | 37.88 | 0.40 | 95.48 | 8.00 | 31.63 | 0.89 | 35.41 |
| Burn S | Dis | 12.00 | 46.75 | 1.29 | 36.16 |  | 37.88 | 0.40 | 95.48 |  | 31.63 | 0.89 | 35.41 |

Abbreviations: ICU – intensive care unit; G – group room; S – single bed room; Dis – discharge; tran. – transfer; prob. – Probability; LOS – length of stay;

# Results

The sensitivity analysis showed when plausible alternative values for critical parameters were used in the model (Table S9). The variation in patients with CRAB, estimates of cost savings and QALYs gained were primarily due to variation from the stochastic outbreaks. There was a noticeable decrease in cost savings with a higher cost of metagenomics.

When simulations without a CRAB outbreak were removed (~20% of iterations) from the analysis, the percentage of cost-effective iterations increased from 58% to 61% and 60% to 64% for scenario 1 vs 2 and scenario 1 vs 3 respectively (Table S10)

Figure S2: Cost breakdown for each scenario


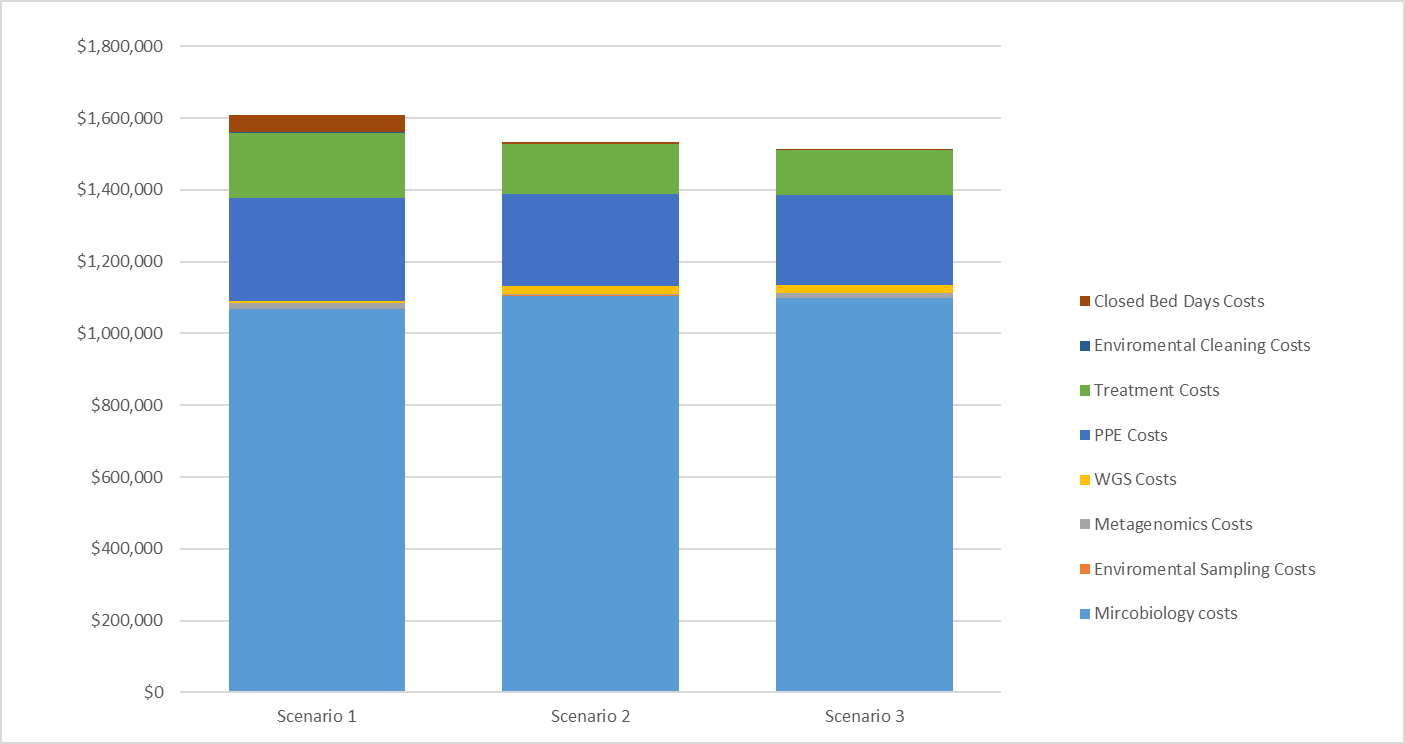
Abbreviations: PPE – personal protective equipment; WGS – whole genome sequencing;

Figure S3: Scatterplot of incremental costs and QALYs (all patients) for a) Scenario 2 versus Scenario 1.


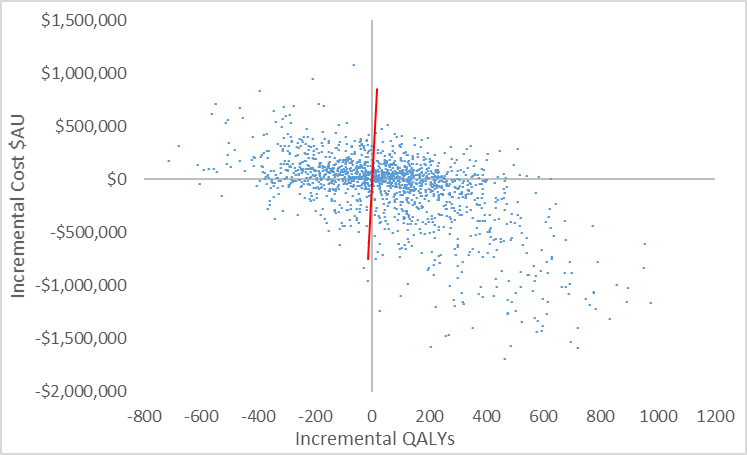


Note: Each dot represents an incremental cost and incremental QALY pairing, using the assigned distributions around each model parameter, selected randomly during 5000 iterations. Dots falling below the diagonal line (the willingness-to-pay threshold of AU$50,000 per QALY) are considered cost-effective. The proportion of simulations considered cost-effective was 57.4%. Abbreviation: QALYs – Quality adjusted life years

**Table S9: One-way sensitivity analyses^1^ of model parameters by Scenario**

|  | **Number of transmissions** | | | **Costs** | | | **QALY** | | | **S1 vs S2**  **Incremental** | | | **S1 vs S3**  **Incremental** | | |
| --- | --- | --- | --- | --- | --- | --- | --- | --- | --- | --- | --- | --- | --- | --- | --- |
|  | **S1** | **S2** | **S3** | **S1** | **S2** | **S3** | **S1** | **S2** | **S3** | **Cost** | **QALY** | **CE%** | **Cost** | **QALY** | **CE%** |
| **Base case** | 29.73 | 15.25 | 11.37 | $1,608,571 | $1,533,471 | $1,514,748 | 6,578 | 6,637 | 6,652 | -$75,099 | 59 | 58% | -$100,604 | 74 | 60% |
| **Cost of WGS (base AU$150)** | | | | | |  |  |  |  |  |  |  |  |  |  |
| Cost of WGS = $120 | 30.23 | 15.25 | 11.23 | $1,695,225 | $1,610,757 | $1,591,078 | 6,578 | 6,637 | 6,652 | -$84,468 | 59 | 58% | -$104,147 | 74 | 60% |
| Cost of WGS = $180 | 30.04 | 15.27 | 11.38 | $1,697,496 | $1,620,337 | $1,600,434 | 6,578 | 6,637 | 6,652 | -$77,159 | 59 | 58% | -$97,062 | 74 | 61% |
| **Cost of Metagenomics (base AU$355)** | | | | | |  |  |  |  |  |  |  |  |  |  |
| Cost of MG = $251 | 30.06 | 15.59 | 11.39 | $1,688,701 | $1,613,823 | $1,589,864 | 6,578 | 6,637 | 6,652 | -$74,878 | 59 | 58% | -$98,837 | 74 | 62% |
| Cost of MG = $559 | 30.28 | 15.19 | 11.68 | $1,685,632 | $1,640,340 | $1,610,328 | 6,578 | 6,637 | 6,652 | -$45,291 | 59 | 58% | -$75,304 | 74 | 62% |
| **Mortality rate of CRAB infection (base 0.077)** | | | | | | |  |  |  |  |  |  |  |  |  |
| MR 0.065 | 30.72 | 15.18 | 11.23 | $1,615,711 | $1,533,494 | $1,513,544 | 6,570 | 6,640 | 6,654 | -$82,217 | 70 | 60% | -$102,168 | 84 | 62% |
| MR 0.154 | 29.26 | 14.79 | 11.06 | $1,605,227 | $1,530,003 | $1,512,344 | 6,574 | 6,637 | 6,652 | -$75,225 | 63 | 59% | -$92,884 | 78 | 62% |
| **Environmental swab culture sensitivity (base 0.4)** | | | | | | |  |  |  |  |  |  |  |  |  |
| prob. 0.30 | 29.77 | 16.27 | 11.36 | $1,606,554 | $1,541,232 | $1,515,366 | 6,576 | 6,638 | 6,655 | -$65,322 | 62 | 60% | -$91,188 | 79 | 62% |
| prob.0.46 | 30.11 | 14.50 | 11.17 | $1,612,021 | $1,527,618 | $1,513,797 | 6,576 | 6,641 | 6,655 | -$84,403 | 65 | 60% | -$98,224 | 79 | 61% |
| **Environmental metagenomics sensitivity (base 0.80)** | | | | | | |  |  |  |  |  |  |  |  |  |
| prob.0.60 | 30.20 | 15.96 | 12.38 | $1,612,717 | $1,541,271 | $1,522,314 | 6,575 | 6,633 | 6,653 | -$71,446 | 59 | 58% | -$90,403 | 78 | 61% |
| prob.0.92 | 29.99 | 15.17 | 9.91 | $1,610,563 | $1,532,860 | $1,502,746 | 6,579 | 6,638 | 6,657 | -$77,703 | 59 | 59% | -$107,818 | 79 | 62% |
| **Time return for WGS results (base 7 days)** | | | | | | |  |  |  |  |  |  |  |  |  |
| 3 days | 30.20 | 15.96 | 12.38 | $1,612,717 | $1,541,271 | $1,522,314 | 6,575 | 6,633 | 6,653 | -$71,446 | 59 | 58% | -$90,403 | 78 | 61% |
| 10 days | 29.99 | 15.17 | 9.91 | $1,610,563 | $1,532,860 | $1,502,746 | 6,579 | 6,638 | 6,657 | -$77,703 | 59 | 59% | -$107,818 | 79 | 62% |

Abbreviations: CRAB - Carbapenem-resistant *Acinetobacter baumannii*; WGS – whole genome sequencing; QALY – Quality adjusted life years; S – Scenario; CE – Cost effective at $50,000 per QALY

^1^ Analyses were performed by changing the parameter of interest (±15%) and re-running the model with 5000 Monte Carlo simulations

Table S10: Projected health and economic outcomes over the outbreak by Scenario - outbreak iterations only**^(1)^**

|  | | **Scenario 1**  mean (Q1, Q3) | **Scenario 2**  mean (Q1, Q3) | | **Scenario 3**  mean (Q1, Q3) | **S1 vs S2**  mean diff. (%) | **S1 vs S3** mean diff. (%) |
| --- | --- | --- | --- | --- | --- | --- | --- |
| No. Infections and Colonisations | | | | | | | |
| CRAB ST1050 | 37 (6, 54) | | 19 (4, 24) | 14 (4, 16) | -18 (-49%) | -25 (-64%) |  |
| Total Hospital costs | | $1,662,405 ($1,447,139, $1,762,805) | $1,562,276 ($1,460,144, $1,626,809) | | $1,538,062 ($1,458,889, $1,581,524) | $-100,129 (-6%) | $-124,343 (-7%) |
| No. Deaths | | | | | | | |
| CRAB ST1050 | | 1.8 (0.0, 3.0) | 0.9 (0.0, 1.0) | | 0.7 (0.0, 1.0) | -0.9 (-49%) | -1.1 (-62%) |
| QALYs | | 6,549 (6,448, 6,682) | 6,623 (6,531, 6,723) | | 6,641 (6,552, 6,735) | 74 (1%) | 93 (1%) |
| Cost effectiveness at willingness to pay threshold of $50,00 per QALY gained | | | | |  |  | |
| likelihood is cost effective | | |  | |  | 61% | 64% |

Abbreviations: CRAB - Carbapenem-resistant *Acinetobacter baumannii*; ST1050 – subtype 10050; Q1 – 1^st^ quartile; Q3 – 3^rd^ quartile; QALYs – Quality adjusted life years;

^(1)^  Of the 5000 simulations, only iterations where greater than 3 ST1050 CRAB cases were identified were included.

# References

1. Gurieva T, Dautzenberg MJD, Gniadkowski M, Derde LPG, Bonten MJM, Bootsma MCJ. The Transmissibility of Antibiotic-Resistant Enterobacteriaceae in Intensive Care Units. Clinical Infectious Diseases **2018**; 66(4): 489-93.

2. Diekmann O, Heesterbeek H, Britton T. Mathematical Tools for Understanding Infectious Disease Dynamics. New Jersey, United Stated of America: Princeton University Press, **2013**.

3. Roberts LW, Forde BM, Hurst T, et al. Genomic surveillance, characterisation and intervention of a carbapenem-resistant Acinetobacter baumannii outbreak in critical care. medRxiv **2020**: 2020.08.10.20166652.

4. Viehman JA, Nguyen MH, Doi Y. Treatment options for carbapenem-resistant and extensively drug-resistant Acinetobacter baumannii infections. Drugs **2014**; 74(12): 1315-33.

5. Kwee F, Walker SA, Elligsen M, Palmay L, Simor A, Daneman N. Outcomes in Documented Pseudomonas aeruginosa Bacteremia Treated with Intermittent IV Infusion of Ceftazidime, Meropenem, or Piperacillin-Tazobactam: A Retrospective Study. The Canadian journal of hospital pharmacy **2015**; 68(5): 386-94.

6. Wozniak TM. Clinical management of drug-resistant bacteria in Australian hospitals: An online survey of doctors' opinions. Infection, Disease & Health **2018**; 23(1): 41-8.

7. Network PI. GUIDANCE ON TREATMENT OF CARBAPENAMASE PRODUCING ENTEROBACTERIACEAE. Available at: <https://www.nhstaysideadtc.scot.nhs.uk/Antibiotic%20site/pdf%20docs/UKCPA%20CPE%20Guidance.pdf>. Accessed 16/04/2020.

8. Health SA. Staphylococcus aureus Bacteraemia (SAB) Management Clinical Guideline. Government of South Australia, **2019**.

9. Al-Jashaami LS, DuPont HL. Management of Clostridium difficile Infection. Gastroenterol Hepatol (N Y) **2016**; 12(10): 609-16.

10. Briggs A, Sculpher M, Claxton K. Decision modelling for health economic evaluation: Oxford University Press, **2006**.
